# Multiple Gastrointestinal Complications of Crack Cocaine Abuse

**DOI:** 10.1155/2014/512939

**Published:** 2014-04-15

**Authors:** Neal Carlin, Nhat Nguyen, Joseph R. DePasquale

**Affiliations:** ^1^School of Graduate Medical Education, Seton Hall University, St. Michael's Medical Center, Newark, NJ 07102, USA; ^2^Department of Internal Medicine, St. Michael's Medical Center, Newark, NJ 07012, USA

## Abstract

Cocaine and its alkaloid free base “crack-cocaine” have long since been substances of abuse. Drug abuse of cocaine via oral, inhalation, intravenous, and intranasal intake has famously been associated with a number of medical complications. Intestinal ischemia and perforation remain the most common manifestations of cocaine associated gastrointestinal disease and have historically been associated with oral intake of cocaine. Here we find a rare case of two relatively uncommon gastrointestinal complications of hemorrhage and pancreatitis presenting within a single admission in a chronic crack cocaine abuser.

## 1. Case

HM is a 53-year-old African American male who presented to the emergency department with complaint of right-sided abdominal pain. The patient has a past medical history significant for hypertension controlled with clonidine and amlodipine, as well as polysubstance abuse. He denied alcohol abuse or any significant familial history of malignancy. The patient stated that he was in his normal state of health when he experienced rapid onset of intense abdominal pain and nausea which was worsened by eating, with no associated fevers, chills, vomiting, or diarrhea. However, patient did admit to intermittent heroin use and smoking crack cocaine on a daily basis.

Initial laboratory analysis revealed that the patient had a serum lipase level greater than 2000 u/L, alkaline phosphatase level of 53 IU/L, AST level of 34, ALT level of 25, total bilirubin level of 1.9 mg/dL, hemoglobin level of 12.9, and creatinine level of 4.0 mg/dL. He was admitted to the medical intensive care unit (MICU) with the diagnosis of acute pancreatitis and acute renal failure.

An abdominal ultrasound revealed a normal sized common bile duct of 3.7 mm, without evidence of cholelithiasis or biliary sludge. A lipid panel was done and showed a serum total cholesterol and triglyceride level of 107 mg/dL and 122 mg/dL, respectively. The patient did not have any other risk factors for pancreatitis; therefore it was concluded that the likely etiology of the patient's pancreatitis was secondary to his crack-cocaine abuse.

The patient remained in the MICU hemodynamically stable and afebrile for four days until being transferred to the medical floor with a hemoglobin level of 9.5 gm/dL. One day following transfer the patient had a single large volume melenic bowel movement and was noted to be lethargic and in acute respiratory distress. He was subsequently transferred back to the intensive care unit and found to have a hemoglobin level of 4.8 gm/dL and diagnosed with hypoxic respiratory failure secondary to gastrointestinal bleeding and acute blood loss.

Endoscopic evaluation via esophagogastroduodenoscopy (EGD) revealed several large ulcers of various sizes, ischemic in appearance throughout the stomach and duodenum. Given the fact that the patient did not have any risk factors for peptic ulcer disease and his history of cocaine abuse, it was thought that the patient's ulcers were likely ischemic in nature. At no time prior to the acute blood loss event secondary to gastric ulcer bleeding was the patient ever hypotensive or intubated. Nonetheless, a serum gastrin and* H. pylori* serology were sent and were negative. The patient was placed on intravenous pantoprazole drip, transfused, and remained in the ICU for further monitoring.

HM was eventually discharged to out-patient followup with the gastroenterology team as well as drug counseling service. He returned in approximately one month, substance-free and devoid of any further complications; however, he failed to return for follow-up endoscopic exam.

## 2. Discussion

Cocaine, benzoylmethyl ecgonine, is a crystalline tropane alkaloid that comes from the leaves of the* Erythroxylum coca* plant; a granular crystalline powder, cocaine hydrochloride, that can be smoked, is produced by dissolving the alkaloid in hydrochloride acid. Cocaine acts centrally by inhibition of the presynaptic reuptake of dopamine, norepinephrine, and serotonin causing stimulation of the central nervous system. It inhibits monoaminooxidases and has a direct anticholinergic effect and stimulates alpha adrenergic receptors. This activation of the sympathetic nervous system produces notorious vasoconstriction and ischemia [1].

Smoking cocaine allows for great absorption of the free base form, which does not undergo first-pass hepatic metabolism. Inhaled use allows for extremely rapid rise in plasma concentration to values greater than 900 ng/mL. Peak values (150 to 200 ng/mL) are reached at 30 to 40 minutes after inhalation of 96 mg of crystalline cocaine hydrochloride [2]. While there is a tremendous inconsistency in percent of actual drug product absorbed, owing to varying degrees of diffusion capacity and local vasoconstriction, substance effects can occur within 8–12 seconds, with similar if not more pronounced clinical effects [2–4]. This is in contrast to other routes of consumption where clinical effect may take up to one minute. The rapidity of effect accounts for the violent “rush” that is described by crack cocaine abusers and is additionally responsible for the psychopathology of significant addiction associated with its use [3, 4].

The clinical, short term effects include decreased appetite, pupil dilatation, vasoconstriction, hypertension, and increased energy, heart rate, temperature, and altered mentation. Cocaine use has been related to myocardial infarction, arrhythmias, cerebral vascular accidents, and convulsions [5, 6]. Pulmonary, renal, obstetrical, and psychiatric events have been reported as well as rare incidents of intestinal ischemia, perforation, retroperitoneal fibrosis, and gastric ulcer and sporadic cases of pancreatic involvement [7–10].

Gastrointestinal hemorrhage and intestinal perforations associated with smoking cocaine have been documented and felt to be due to deep gastric ulcerations from the multisystem toxicity of free based crack [2, 6]. Four patients were reported with perforated gastric ulcers due to smoking crack by Kram et al. [11]. Kodali and Gordon reported an upper gastrointestinal bleed secondary to smoking crack cocaine [12]. Cocaine's blockage of norepinephrine reuptake leads to ensuing mesenteric vasoconstriction and focal tissue ischemia which leads more commonly to perforation [6, 10].

Vazquez-Rodriquez et al. reported that a 21-year-old male used insufflated cocaine and developed abdominal pain 48 hours later with an elevated LDH. The diagnosis of acute pancreatitis was made with no other etiology [5]. The mechanism by which cocaine induces changes in the pancreas is unclear but involves its effect on the presynaptic nerve endings by blocking the reuptake of norepinephrine. This increases the norepinephrine in the synaptic cleft and postsynaptic stimulation. Cocaine also has a direct vasoconstriction effect mediated by the flux of calcium across the endothelial cell membrane. It also causes thrombus formation and platelet aggregation and decreases fibrinolytic activity by stimulating plasminogen activator inhibitor activity which may likely have played an alternative role in the ischemic pathology of crack cocaine induced pancreatitis.

This patient exhibits both hematemesis with multiple large ischemic gastric ulcers and acute pancreatitis. Clinicians must be aware of crack cocaine induced gastrointestinal symptomology and the associated pathophysiology, in order to astutely manage the multiple varied and serious complications affiliated with substance abuse.
